# Novel Small Molecule XPO1/CRM1 Inhibitors Induce Nuclear Accumulation of TP53, Phosphorylated MAPK and Apoptosis in Human Melanoma Cells

**DOI:** 10.1371/journal.pone.0102983

**Published:** 2014-07-24

**Authors:** Jennifer Yang, Matthew A. Bill, Gregory S. Young, Krista La Perle, Yosef Landesman, Sharon Shacham, Michael Kauffman, William Senapedis, Trinayan Kashyap, Jean-Richard Saint-Martin, Kari Kendra, Gregory B. Lesinski

**Affiliations:** 1 Department of Internal Medicine, The Ohio State University, Columbus, Ohio, United States of America; 2 Center for Biostatistics, The Ohio State University, Columbus, Ohio, United States of America; 3 Department of Veterinary Biosciences, The Ohio State University, Columbus, Ohio, United States of America; 4 Karyopharm Therapeutics, Natick, Massachusetts, United States of America; University of Queensland Diamantina Institute, Australia

## Abstract

XPO1/CRM1 is a key nuclear exporter protein that mediates translocation of numerous cellular regulatory proteins. We investigated whether XPO1 is a potential therapeutic target in melanoma using novel selective inhibitors of nuclear export (SINE). *In vitro* effects of SINE on cell growth and apoptosis were measured by MTS assay and flow cytometry [Annexin V/propidium iodide (PI)], respectively in human metastatic melanoma cell lines. Immunoblot analysis was used to measure nuclear localization of key cellular proteins. The *in vivo* activity of oral SINE was evaluated in NOD/SCID mice bearing A375 or CHL-1 human melanoma xenografts. SINE compounds induced cytostatic and pro-apoptotic effects in both *BRAF* wild type and mutant (*V600E*) cell lines at nanomolar concentrations. The cytostatic and pro-apoptotic effects of XPO1 inhibition were associated with nuclear accumulation of TP53, and CDKN1A induction in the A375 cell line with wild type TP53, while pMAPK accumulated in the nucleus regardless of *TP53* status. The orally bioavailable KPT-276 and KPT-330 compounds significantly inhibited growth of A375 (p<0.0001) and CHL-1 (p = 0.0087) human melanoma cell lines *in vivo* at well tolerated doses. Inhibition of XPO1 using SINE represents a potential therapeutic approach for melanoma across cells with diverse molecular phenotypes by promoting growth inhibition and apoptosis.

## Introduction

Melanoma is the most deadly form of skin cancer, with an estimated 76,100 new cases and 9,710 deaths in the United States alone in 2014 [1]. The incidence of melanoma is rising faster than that of any other cancer, and approximately 232,000 new cases will be diagnosed each year worldwide [2]. Recent therapeutic approaches including small molecule inhibitors of activated BRAF pathways (vemurafenib, dabrafenib) and immunomodulatory agents represent significant advances in melanoma therapy [3], [4]. Although these approaches elicit complete, durable responses in a subset of melanoma patients, many patients develop resistance, or are unable to tolerate adverse events associated with administration of these agents. The genetic and phenotypic heterogeneity of melanoma cells increases the likelihood for the emergence of drug-resistant clonal cell populations and eventually disease recurrence [5]. Such resistance mechanisms could be attributed to the fundamental ability of malignant cells to inactivate tumor suppressor pathways and bypass cell cycle checkpoints. One predominant means by which these regulatory pathways are rendered ineffective is through the inappropriate localization of tumor suppressor (TSP) and growth regulatory proteins (GRP) in the cytoplasm [6], [7], [8]. This process, termed ‘nuclear export’, is gaining attention as a novel therapeutic target that can be inhibited to promote re-activation of tumor suppressive pathways.

**Figure 1:**
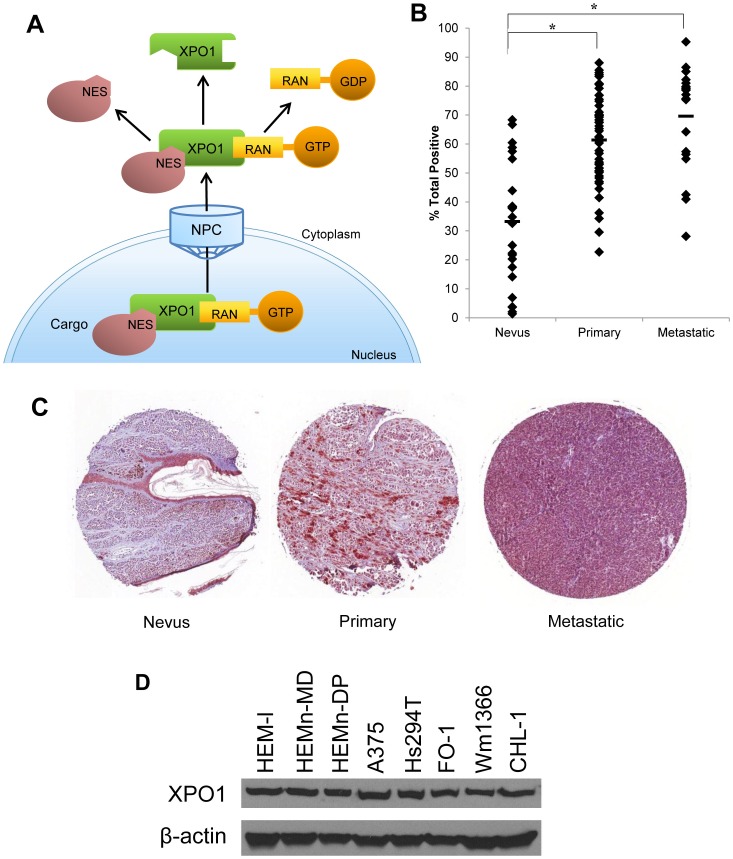
The mechanism of XPO1 export, and its expression in human skin samples and melanoma cell lines. *A*. Mechanistic explanation of XPO1 mediated nuclear export pathway adapted from Kau 2013 [10]. *B*. A human melanoma tissue microarray was stained for XPO1 and quantified. The core includes 56 cases of primary melanoma, 20 cases of metastatic melanoma, and 24 nevus samples. The average percent total XPO1 staining includes weak, medium, and strong positive percentage as determined by the ImageScope software. The nevus, primary melanoma, and metastatic samples were 33.2±20.4, 61.4±14.9, and 69.6±17.5 percent positive for XPO1, respectively. * =  The differences in the level of XPO1 staining between nevus and primary samples, or between nevus and metastatic samples both had p-values of <0.0001.*C*. Representative pictures of skin sample cores. Slides were scanned in at 40× magnification and individual cores taken at 8x using an Aperio ImageScope. *D*. Immunoblot analysis of XPO1 protein expression in a panel of human metastatic melanoma cell lines (A375, Hs294T, FO-1, Wm1366, CHL-1) and three primary human epidermal melanocyte cell lines (HEM-l, HEMn-MP, HEMn-DP). β-actin served as control for equal loading.

One potential target, called Exportin 1 (XPO1, also known as chromosome region maintenance 1, CRM1), belongs to the Karyopherin β family of proteins. XPO1 is one of seven known nuclear export proteins that is known to mediate the specific export of many eukaryotic proteins and certain RNAs by recognizing canonical leucine-rich nuclear export sequences (NES) [9]. Upon binding to RanGTP (ras-related nuclear protein guanosine-5′-triphosphate), XPO1 forms a complex with the nuclear export cargo and is then translocated from the nucleus to the cytoplasm through a passage known as the nuclear pore complex (NPC). Once the complex is in the cytoplasm, RanGTP is hydrolyzed to the inactive RanGDP (ras-related nuclear protein guanosine-5′-diphosphate) and the cargo dissociates from XPO1 where it remains localized to the cytoplasm [10] (Fig. 1A). Despite the existence of seven nuclear export proteins, XPO1 is the *sole* mediator of nuclear export for many cell regulatory proteins including the TP53 and CDKN1A (cyclin-dependent kinase inhibitor 1A), TSP, [11], [12], [13], [14], and mitogen activated protein kinase (MAPK, or extracellular signal-regulated kinase, ERK) [15]. The regulation of diverse cellular pathways presents XPO1 as an attractive therapeutic target, while the non-redundant nature of the pathway may prevent the emergence of drug resistance.

Novel selective inhibitors of nuclear export (SINE) targeting XPO1 are being explored as potential therapeutic approaches for treatment of malignancy. Indeed, XPO1 levels are often elevated in tumors when compared with non-malignant cells of the same lineage, including pancreatic cancer, glioma, and cervical cancer [7], [16], [17]. Importantly, elevated XPO1 expression is generally correlated with poor prognosis in these cancers, as well as in osterosarcoma and ovarian cancer [7], [13], [16], [17], [18], [19]. It is thought that XPO1 may support the malignant phenotype by promoting the export of TSP and GRP out of the nucleus. The non-drug-like, natural product leptomycin B (LMB) has been used to potently inhibit XPO1 function and induce anti-proliferative activity in a range of tumor cell lines, including melanoma [8], [20], [21], [22]. This compound is a potent, fully irreversible inhibitor of XPO1 with a novel mechanism of action [23]. However, due to a very poor therapeutic window in animals [24] and dose-limiting emesis, diarrhea and asthenia with lack of therapeutic efficacy observed in a phase I clinical trial of intravenous LMB [25], no further trials were conducted using this toxic agent. Recent studies also implicate that XPO1 inhibitors may synergize with BRAF inhibition in human melanoma cell lines [26], supporting the concept that nuclear export inhibition may play a role as a therapeutic strategy for this disease. In the present study, we demonstrated that XPO1 expression was elevated in patient primary and metastatic melanomas as compared to nevi. Therefore we hypothesized that inhibition of XPO1 in human melanoma cells would induce nuclear retention of key proteins that promote tumor suppressive pathways and inhibit melanoma cell viability. Treatment of the cells was associated with increased nuclear retention of TP53 and pMAPK, cell cycle arrest, and apoptosis. We utilized a novel platform of SINE compounds that demonstrated significant anti-tumor activity both *in vitro* and using *in vivo* xenograft models of melanoma. These data suggest that small molecule XPO1 inhibitors represent a novel therapeutic approach for melanoma and potentially other malignancies.

## Materials And Methods

### Drugs

Selective Inhibitor of Nuclear Export (SINE) compounds, a family of small drug-like molecules, were provided by Karyopharm Therapeutics, Inc. (Natick, MA). SINE compounds show extremely high selectivity for blocking XPO1 without any significant effects in standard protein screens (including other cysteine-active kinases, caspases and other enzymes), cytochrome P450s, or the hERG ion channel (personal communication, Karyopharm Therapeutics, Inc.). KPT-185 or the 10-100X less active (as an XPO1 inhibitor) trans-isomer (KPT-185-trans) were resuspended in DMSO at stock concentrations of 15.48 mM and 12.66 mM respectively. KPT-276 and KPT-330 were resuspended in DMSO at stock concentrations of 18.77 mM or 18.05 mM respectively. For *in vivo* studies, KPT-276 was suspended to a 7.5 mg/mL concentration and KPT-330 to a 1.5 mg/mL with 0.6% w/v Pluronic F-68 and 0.6% w/v PVP K-29/32 in water as the diluent. Etoposide was from Sigma (E-1383), resuspended in DMSO to a stock concentration of 20 mM.

### Cell lines

The A375, Hs294T, and CHL-1human metastatic melanoma cell lines were obtained from the American Type Culture Collection (Manassas, VA). The Wm1366 human melanoma cell line was obtained from the Wistar Collection as a gift from Dr. Meenhard Herlyn (Wistar Institute, Philadelphia, PA). The FO-1 human melanoma cell line was a gift from Dr. Soldano Ferrone (Massachusetts General Hospital, Boston, MA) [27]. The Human Epidermal Melanocytes-light (HEM-l) primary cells were purchased from ScienCell (Carlsbad, CA). Two additional Human Epidermal Melanocyte primary cells: neonatal, Moderately Pigmented (HEMn-MP) and Darkly Pigmented (HEMn-DP) were purchased from Life Technologies (Carlsbad, CA).

### Cellular growth assay

Cells were seeded in 96-well plates (2×10^4^) in triplicate and allowed to adhere overnight. Fresh media containing KPT-185, KPT-185-trans, KPT-276, or KPT-330 was added to each well at various concentrations (range  = 40–10240 nM) and cells were incubated at 37°C/5% CO_2_ for 72 hours. Cells treated with DMSO (diluent) and untreated cells served as controls. At this time, the percentage of cell growth was evaluated using the MTS assay (CellTiter 96 AQueous Non-Radioactive Cell Proliferation Assay, Promega) and quantified by determining the optical density at 595 nm using a Bio-Rad iMark™ microplate reader.

### Analysis of apoptosis via Annexin V/Propidium Iodide (PI) staining

Phosphatidyl serine exposure was assessed in tumor cells and melanocytes by flow cytometry using APC-Annexin V and propidium iodide (PI; BD Pharmingen, San Diego, CA) as previously described [28]. Each analysis was performed utilizing at least 10,000 events. We considered the Annexin V positive cells (early apoptosis) and Annexin V/PI double positive cells (late apoptosis) cells as the apoptotic cell population.

### Cell cycle assay

Cell-cycle analysis was conducted using propidium iodide (PI) following the manufacturer's protocol (BD Pharmigen). Cells were plated in 6-well plates and treated for 24 h or 48 hr with 1 µM of KPT-185. A total of 1×10^5^ cells per analysis were examined by flow cytometry (FACSCalibur), and analyzed using ModFit LT 3.3.

### Subcellular fractionation assay

Human melanoma cell lines were seeded in 6-well plates (2×10^5^) and allowed to adhere overnight. After treatment for various time points, the cells were harvested then fractionated using NE-PER Nuclear and Cytoplasmic Extraction Reagents from Thermo Scientific following the manufacturer's protocol. Nuclear and cytoplasmic fractions were validated via immunoblot analysis using Lamin B and GAPDH antibodies.

### Western blot analysis

Immunoblots were prepared as previously described and probed with antibodies specific for TP53, CDKN1A (p21), XPO1, HDM2 (MDM2), Lamin B (Santa Cruz Biotechnology, Santa Cruz, CA), pMAPK, PARP (Cell Signaling Technology), GAPDH or β-actin (Sigma) [29]. Following incubation with the appropriate horseradish peroxidase-conjugated secondary antibody, immune complexes were detected using the ECL Plus detection kit (Amersham Biosciences, Ayesbury, UK).

### Murine tumor models and treatments

All animal studies were conducted under a protocol (2009A0178-R1) approved by The Ohio State University Institutional Animal Care and Use Committee (IACUC). A subcutaneous (s.c.) tumor model of melanoma was employed to determine whether treatment with the orally bioavailable SINE compounds, KPT-276 (preclinical tool compound) or KPT-330 (currently in Phase 1 studies in humans with advanced cancers), would be effective in decreasing tumor growth *in vivo*. Female NOD/SCID mice (n = 6 mice/group; 6-8 weeks of age; Jackson Laboratory, Bar Harbor, ME) were injected subcutaneously (s.c.) in the right flank with 2×10^6^ human A375 or CHL-1 melanoma cells (Day 0). Once tumors were palpable, mice were randomized to one of two treatment groups: [a] diluent, [b] KPT-276 (75 mg/kg), or [c] KPT-330 (15 mg/kg). All treatments were administered via oral gavage in a volume of 200 µL three times weekly (Monday, Wednesday, Friday). Bi-dimensional tumor measurements were obtained three times weekly using microcalipers. Mice were euthanized and tumors were collected from all animals once diluent-treated tumors reached a volume of 1500 mm^3^. For histology studies, a subset of animals (n = 2–3/group) were treated with diluents, KPT-276, or KPT-330 for one week, after which tumors were collected to avoid necrosis. The weights of all animals receiving diluent, KPT-276 or KPT-330 were monitored for dosing and to assess toxicity throughout the study.

### Immunohistochemical staining

For analysis of XPO1 expression in human samples, an unstained (5 µm) slide of a commercially available tissue microarray (1 mm cores) containing 56 cases of primary melanoma, 20 cases of metastatic melanoma, and 24 cases diagnosed as nevus (US Biomax, Inc., Catalog #ME1004a) was subjected to immunohistochemical analysis using an antibody targeting XPO1 (Santa Cruz H-300, sc-5595, lot # D0110) according to manufacturer's recommendations (1∶50). For mouse studies, tumor xenografts were fixed in formalin, embedded in paraffin, a sectioned at 4 µm with two sections per slide. Slides were deparaffinized, rehydrated, then stained with anti-TP53 (Catalog # M7001, Clone DO-1, Dako), CDKN1A (Santa Cruz, Catalog# sc-397), Ki67 (Catalog # M7240, Clone MIB-1, Dako), or anti-pMAPK (Cell Signaling Technology, Catalog # 4370) antibodies using the Dako Autostainer Immunostaining System, with Vulcan Fast Red™ or Biocare Romulin AEC chromogens and counterstained with Richard Allen hematoxylin. All samples were analyzed in a blinded fashion by an experienced pathologist (Dr. Krista La Perle). Automatic quantification of immunoreactivity in viable portions of xenograft tissue samples and tissue cores segmented with the TMA Lab (Aperio Technologies, Vista, CA) was performed on whole slide images scanned at 40× magnification (ScanScope XT, Aperio Technologies) using the Positive Pixel Count Algorithm in the ImageScope software, as well as the Color Deconvolution and Cytoplasmic algorithms available in the Image Analysis Toolbox (Aperio Technologies).

### Statistical analysis

The 4-parameter logistic or Hill model [30] was the assumed dose-response relationship for the *in vitro* inhibition and cell viability experiments. Nonlinear least squares regression was used to estimate the model parameters and generate 95% confidence intervals for the IC_50_ estimates. For *in vivo* experiments, tumor volumes were first log-transformed to meet test assumptions of normality and homoscedasticity. Changes in tumor volumes were analyzed using a two-sample t-test to evaluate the difference from first day of treatment to the last for A375 xenografts (day 17), and from day 1 to day 10 for CHL-1 xenografts as day 10 marks the first mouse sacrificed when 1500 mm^3^ tumor volume was reached. ANOVA was used to compare the expression of XPO1 staining among samples from the melanoma tissue core array. The Wilcoxon rank-sum test was used to analyze data comparing growth inhibition and apoptosis of the five metastatic melanoma cell lines to the three melanocyte cell lines.

### Ethics statement

This study was carried out with approval by the Ohio State University IACUC, protocol number 2009A0178-R1. Tumor cell injections were performed under isoflorane anesthesia, and all efforts made to minimize suffering. Two methods of euthanasia were utilized, with carbon dioxide asphyxiation followed by cervical dislocation.

## Results

### XPO1 protein is expressed in human melanoma tissue samples and cell lines

To determine the potential of XPO1 as a molecular target in melanoma cells, XPO1 protein expression was characterized in a human melanoma tissue microarray (nevi, primary melanomas, and metastatic melanomas), a panel of human metastatic melanoma cell lines (A375, Hs294T, FO-1, Wm1366, CHL-1) or three primary human epidermal melanocyte cell lines (HEM-l, HEMn-MP, HEMn-DP). The total percentage of cells staining positive for XPO1was significantly higher in both primary melanoma (p<0.0001) and metastatic melanoma (p<0.0001) when compared to tissues from nevi (Fig. 1B–C). XPO1 protein was also consistently expressed in a panel of five human metastatic melanoma cell lines, and in three primary human melanocyte cell lines (Fig. 1D). Abundant XPO1 expression was observed both in cell lines harboring a *BRAF V600E* mutation (A375, Hs294T, FO-1) and those cell lines with wild type (WT) *BRAF* (Wm1366, CHL-1). This panel of cell lines was diverse with respect to other mutations relevant to melanoma, including *TP53*, *NRAS*, *CDKN2A*, and *PTEN* (Table 1).

**Table 1:** Mutational status of key genes in the panel of human melanoma cell lines.

	A375	Hs294T	FO-1	Wm1366	CHL-1
**BRAF**	p.V600E (−/−)	p.V600E (−/−)	p.V600E (−/−)	WT	WT
**CDKN2A**	p.E61* (−/−)	N/A	N/A	N/A	p.W100*
	p.E69* (−/−)				
**NRAS**	WT	WT	WT	p.Q61L	WT
**PTEN**	WT	N/A	p.G129R (−/−)	WT	WT
**TP53**	WT	p.P72R (−/−)	p.P72R (−/−)	p.E258K (−/−)	p.H193R (−/−)

* =  mutation results in stop codon. (+/−)  =  heterozygous. (−/−)  =  homozygous mutated.

### KPT-185, a novel XPO1 inhibitor induces cytostatic effects in melanoma cells

**Figure 2:**
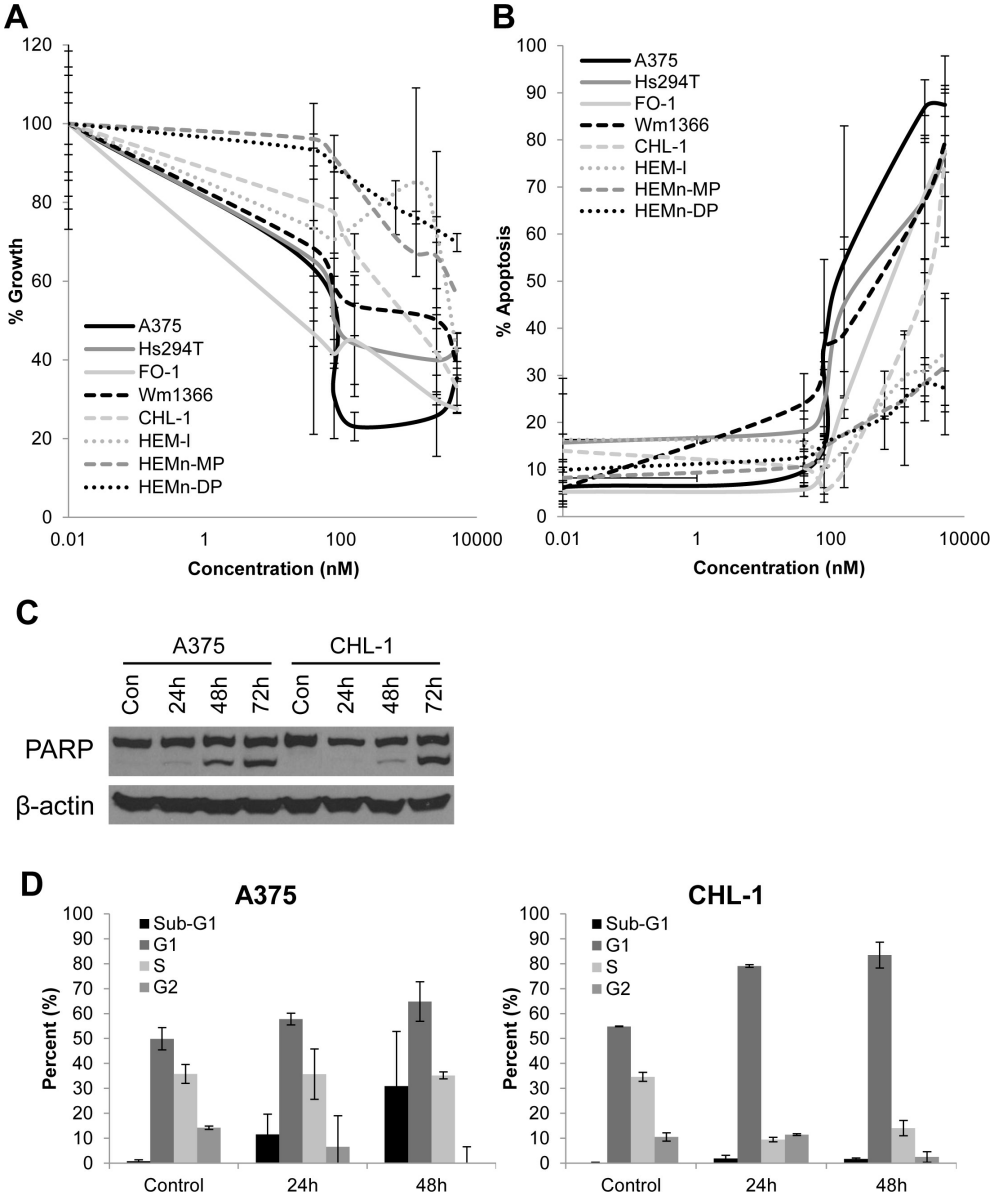
KPT-185 induces cytostatic and cytotoxic effects in melanoma cells. *A*. The cytostatic effect of KPT-185 was evaluated in a panel of human melanoma and melanocyte cell lines after 72 hours of treatment with a range of concentrations (0–10.24 µM) using an MTS assay. *B*. Following procedure as in *A*, the cytotoxic effect of KPT-185 was evaluated in the same panel of cell lines via Annexin V/PI staining. The contrast in sensitivity between the five melanoma lines and the 3 melanocyte lines are significantly different (p = 0.0253). Error bars for both part *A* and *B* represent standard deviations from at least two independent biologic replicates. *C*. Lysates were collected at indicated time points after treatment with KPT-185 at the IC_50_ for apoptosis for each cell line and assessed via immunoblot analysis. β-actin serves as loading control. *D*. Cell cycle analysis was performed to dissect the cytostatic readout observed in *A*. Cells were treated with KPT-185 at 1 µM for 24 h or 48 hr, and then stained with PI. Error bars represent standard deviations from two independent biologic replicates.

XPO1 has been shown in other malignant cell lines to export several cell-cycle regulatory proteins, including TP53, pMAPK and the CDKN1A cyclin-dependent kinase inhibitor [11], [12], [13], [14]. The known association of these growth regulatory proteins with XPO1 prompted us to first examine how inhibition of XPO1 would alter melanoma cell growth. A panel of human metastatic melanoma cell lines were treated with varying concentrations of the KPT-185 XPO1 inhibitor (Range  = 0–10.24 µM) or DMSO (vehicle) as a negative control. Significant inhibition of cell growth was observed in all melanoma cell lines following a 72 hour treatment with KPT-185 as compared to control (Fig. 2A). The response of individual melanoma cell lines to KPT-185 was somewhat variable as concentrations in the 50 nM range induced 50% growth inhibition in a subset of cell lines (A375 and FO-1), while higher, but physiologically-achievable concentrations of drug (591.6 nM) were required for cytostatic effects in others (CHL-1). The IC_50_ for the panel of cell lines is summarized in Table 2. HEM-1 cells were less sensitive to KPT-185 treatment as compared to most melanoma cell lines, with an IC_50_ of 4129.3 nM after a 72 hour treatment. Neither HEMn-MP nor HEMn-DP cell lines were able to achieve IC_50_ even at the highest concentration of 10240 nM. The sensitivity of the 5 melanoma lines and the 3 melanocyte lines to KPT-185 treatment were significantly different when compared even at high drug concentrations (p = 0.0253) (Table 2).

**Table 2:** The IC_50_ values of KPT185 for growth inhibition and apoptosis in a panel of human melanoma cell lines.

	Growth Inhibition		Apoptosis	
Cell line	IC50 (nM)	95% CI (nM)	IC50 (nM)	95% CI (nM)
A375	45.9	34.6–57.2	146.7	101.9–191.4
Hs294T	48.9	28.6–69.3	212.2	110.6–313.8
FO-1	32.1	0.0–77.0	296.6	212.9–380.2
Wm1366	346.9	0–794.2	660.9	271.7–1050.2
CHL-1	591.6	176.6–1006.5	2414.1	2271.4–2556.7
HEM-l	4129.3	3050.0–5208.5	3170.0	898.0–5441.9
HEMn-MP	Not achieved	N/A	Not achieved	N/A
HEMn-DP	Not achieved	N/A	Not achieved	N/A

A panel of human melanoma cell lines (A375, Hs294t, FO-1, Wm1366, CHL-1) and three normal human melanocyte cell lines (HEM-l, HEMn-MP, HEMn-DP) were treated with a range of KPT-185 concentrations (0–10.24 µM). After 72 hr of treatment, growth inhibition was evaluated using an MTS assay, and apoptosis was evaluated via Annexin V/PI staining and flow cytometric analysis. The 5 melanoma lines and the 3 melanocyte lines had significantly different sensitivity (p = 0.0253) to KPT-185 treatment.

### KPT-185 induces apoptosis of melanoma cells

To assess whether the growth inhibitory effects of KPT-185 were due in part to decreased cell viability, the same panel of human melanoma cells was stained with Annexin V/Propidium Iodide (PI) following a 72 hour treatment with various concentrations of the KPT-185, and analyzed via flow cytometry. All human melanoma cell lines were sensitive to the pro-apoptotic effects of KPT-185, to a variable degree. Consistent with our cell proliferation data (Table 2 and Fig. 2B), cell lines with WT *BRAF* typically required a higher concentration of KPT-185 in order to induce a comparable pro-apoptotic effect *in vitro*. However, these data were obtained in a somewhat limited panel of cell lines with inherent genetic variability (Table 1). HEM melanocyte cell lines also were less sensitive to the pro-apoptotic effects of KPT-185 as compared to a majority of melanoma cell lines, with an IC_50_ of 3170.0 nM for HEM-l treated with KPT-185 and not achievable for either HEMn-MP or HEM-DP (Table 2). Similar to prior studies utilizing LMB [22], KPT-185 treatment of melanoma cell lines led to cleavage of PARP, indicative of apoptotic cell death (Fig. 2C). Further analysis indicated that A375 and CHL-1 cells arrested in the G1 phase of the cell cycle following treatment with KPT-185, accompanied by a sub-G1 population consistent with apoptosis (Fig. 2D). These data were congruent with a prior study using a different nuclear export inhibitor (KPT-251), where the authors observed G1 accumulation with inhibitor treatment [26].

### XPO1 inhibition with KPT-185 induced nuclear localization of TP53, pMAPK and CDKN1A accumulation

**Figure 3:**
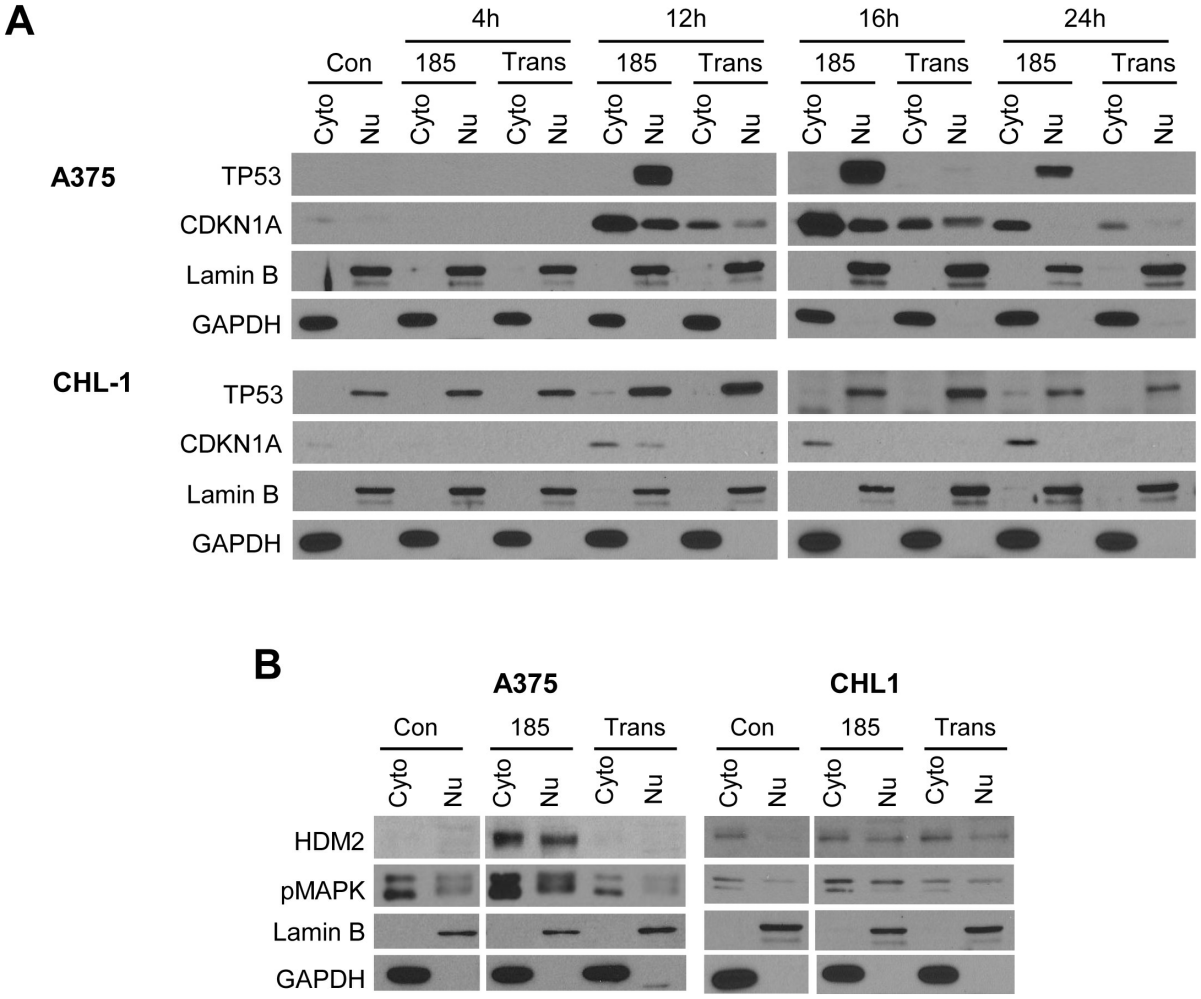
KPT-185 upregulates TP53 and CDKN1A, and promotes their differential protein expression and localization. A375 is *TP53* WT and CHL-1 has *TP53* mutations (H193R (−/−) and E258K). *A*. Cells were treated with vehicle alone, 500 nM of KPT-185 or KPT-185-trans as a control. Samples were collected and fractionated at 4, 8, 12, 16, and 24 hr and evaluated via immunoblot analysis. *B*. Cells were treated with vehicle, KPT-185 at the respective IC_50_ concentrations. Lysates were collected at 16 hr. Subcellular fractionation was used to assess differences in protein localization via immunoblot analysis. Lamin B and GAPDH serve as loading controls for nuclear and cytoplasmic compartments respectively. The data shown are representative of 2 to 3 independent biological experiments.

To gain greater insight into the mechanisms by which KPT-185 induced apoptosis in melanoma cell lines with unique molecular profiles, the nuclear and cytoplasmic localization of known XPO1 cargo proteins was next assessed. Although multiple mechanisms of death are likely operative in response to these agents, nuclear accumulation of these proteins serves as an informative biomarker validating drug action. The TP53 protein is a classical tumor suppressor protein (TSP), and accumulation of pMAPK in the nucleus has been associated with apoptosis [22], [31]. Both of these proteins are targets of XPO1-mediated nuclear export. We measured the nuclear accumulation of TP53 following XPO1 inhibition in lysates from representative melanoma cells via immunoblot analysis. For these studies, two melanoma cell lines with varying mutational status of *BRAF* and *TP53* were utilized: A375 (*BRAF V600E* mutant, *TP53* WT), and CHL-1 (*BRAF* wild type, *TP53* homozygous mutant; H193R). Interestingly, the CHL-1 cell line was less sensitive to KPT-185-induced growth inhibition (Table 2 and Fig. 2A) and apoptosis (Table 2 and Fig. 2B). Cells were collected at 4, 12, 16, and 24 hour time points and fractionated into cytoplasmic and nuclear compartments following treatment with KPT-185. This was done to capture the modulatory effect of XPO1 inhibition on the localization and expression of key tumor suppressor proteins prior to apoptosis. In these studies, KPT-185 treatment upregulated the expression of TP53 protein in A375 cells and nuclear localization at the 16 hour time point. Consistent with these data, downstream upregulation of CDKN1A was also observed in this cell line following treatment with KPT-185 (Fig. 3A). CDKN1A prevents the cells from proliferating by inhibiting cyclin D1, which cooperates with the accumulation of cells in G1 observed in cell cycle analysis (Fig. S1D). In contrast to A375 cells, the CHL-1 cell line which contains mutated *TP53* appeared to have only limited TP53 or CDKN1A induction with KPT-185 treatments (Fig. 3A).

Etoposide was also used in parallel to compare the activity of KPT-185 to a known TP53 upregulating stimulus. This approach allowed us to verify the functionality of TP53 in each cell line utilized (Fig. S1A). Both etoposide and KPT-185 induced TP53 upregulation and downstream activation of CDKN1A, and robust accumulation of both proteins in the nucleus. In addition to these characteristic features of TP53 upregulation, inhibition of nuclear export led to an increased entrapment of pMAPK within the nucleus (Fig. S1A) but did not have observable effect on the overall expression of pMAPK (Fig. S1B). Due to the ability of KPT-185 to upregulate TP53, we next examined whether loss of HDM2, represented a mechanism by which TP53 induction occurred. Indeed, when TP53 expression is elevated, it can promote the expression of HDM2 which binds and catalyzes the ubiquitylation and subsequent protesomal degradation of TP53 [32]. In the A375 cell line, we observed robust expression of HDM2 in both the cytoplasmic and nuclear compartment following KPT-185 treatment, where there was no detectable protein at basal level (Fig. 3B). This corresponds with the ability of TP53 to induce the expression of HDM2 in a part of an autocrine feedback loop, but suggested degradation of HDM2 in response to KPT-185 is not the mechanism by which TP53 is upregulated [33]. In agreement with a prior study using LMB [22], phosphorylated MAPK also accumulated in the nuclear compartment of the A375 and CHL-1 melanoma cell lines following KPT-185 treatment (Fig. 3B). These effects were less prominent in cells treated with the KPT-185-trans compound (10-100X less active as an XPO1 inhibitor than the cis-compound KPT-185) which has reduced XPO1 inhibition activity (Fig. 3).

### KPT-SINE treatments inhibited melanoma growth *in vivo*

**Figure 4:**
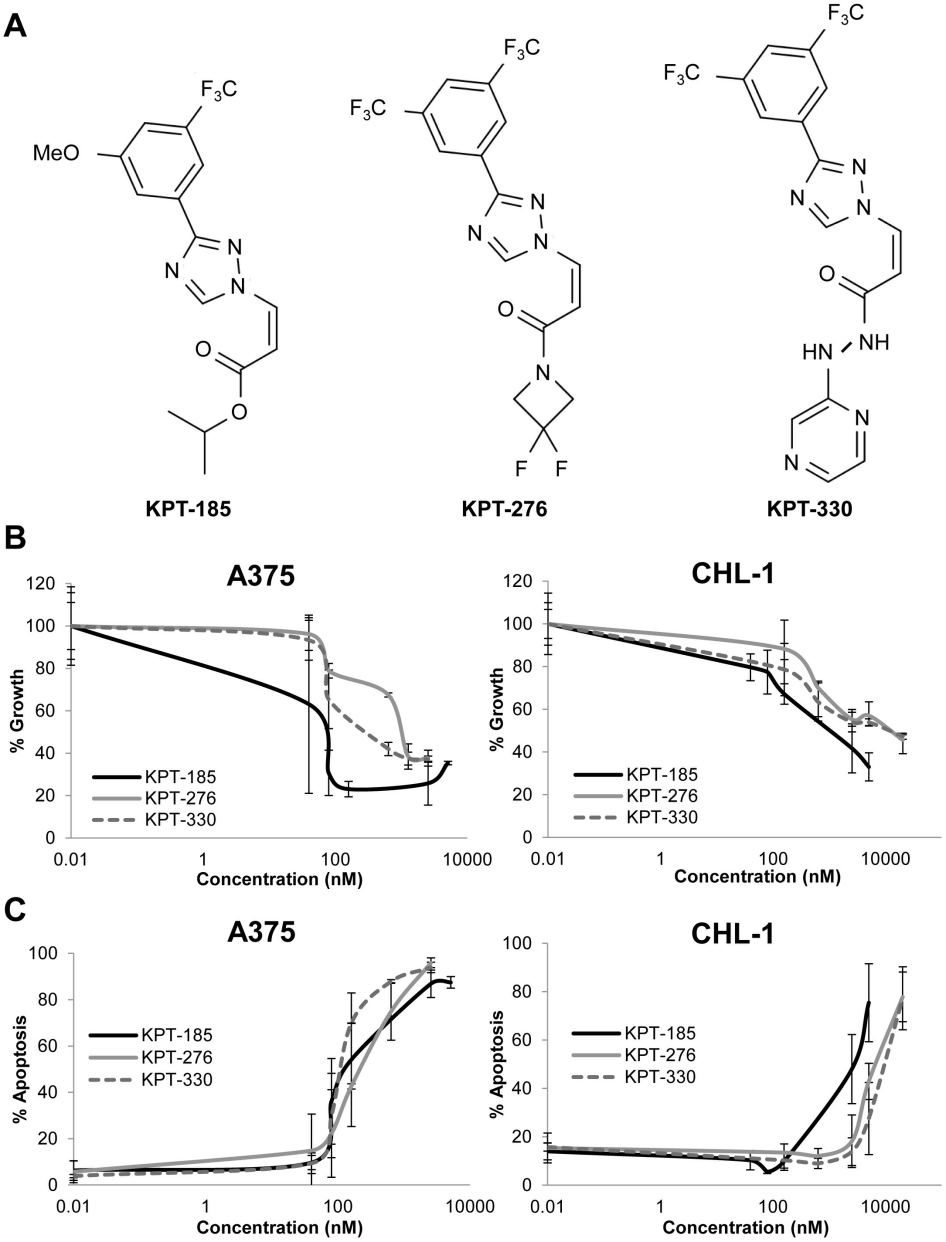
KPT-276 and KPT-330 induce cytostatic effects in melanoma cells. *A*. The chemical structures of KPT-185, KPT-276, and KPT-330. *B*. The cytostatic effect of KPT-276 was evaluated in a panel of human melanoma cell lines after 72 hours of treatment with a range of concentrations (0−10.24 µM) using an MTS assay. The IC_50_ value of A375 for KPT-276 was 320.6 nM and the IC_57_ value was 3879.4 nM for CHL-1. For KPT-330, the IC_50_ values were 119.9 nM and 7533.8 nM for A375 and CHL-1, respectively. *C*. The pro-apoptotic effect of KPT-276 was evaluated under the same parameters as in part *B*. The IC_50_ values were 232.8 nM and 6129.4 nM for A375 and CHL-1, respectively. For KPT-330, the IC_50_ values were 112.3 nM and 7692.5 nM for A375 and CHL-1, respectively. Values on KPT-185 were taken from Figure S1 and graphed with KPT-276 and KPT-330 for comparison. Error bars represent standard deviations from at least two independent biologic replicates.

**Figure 5:**
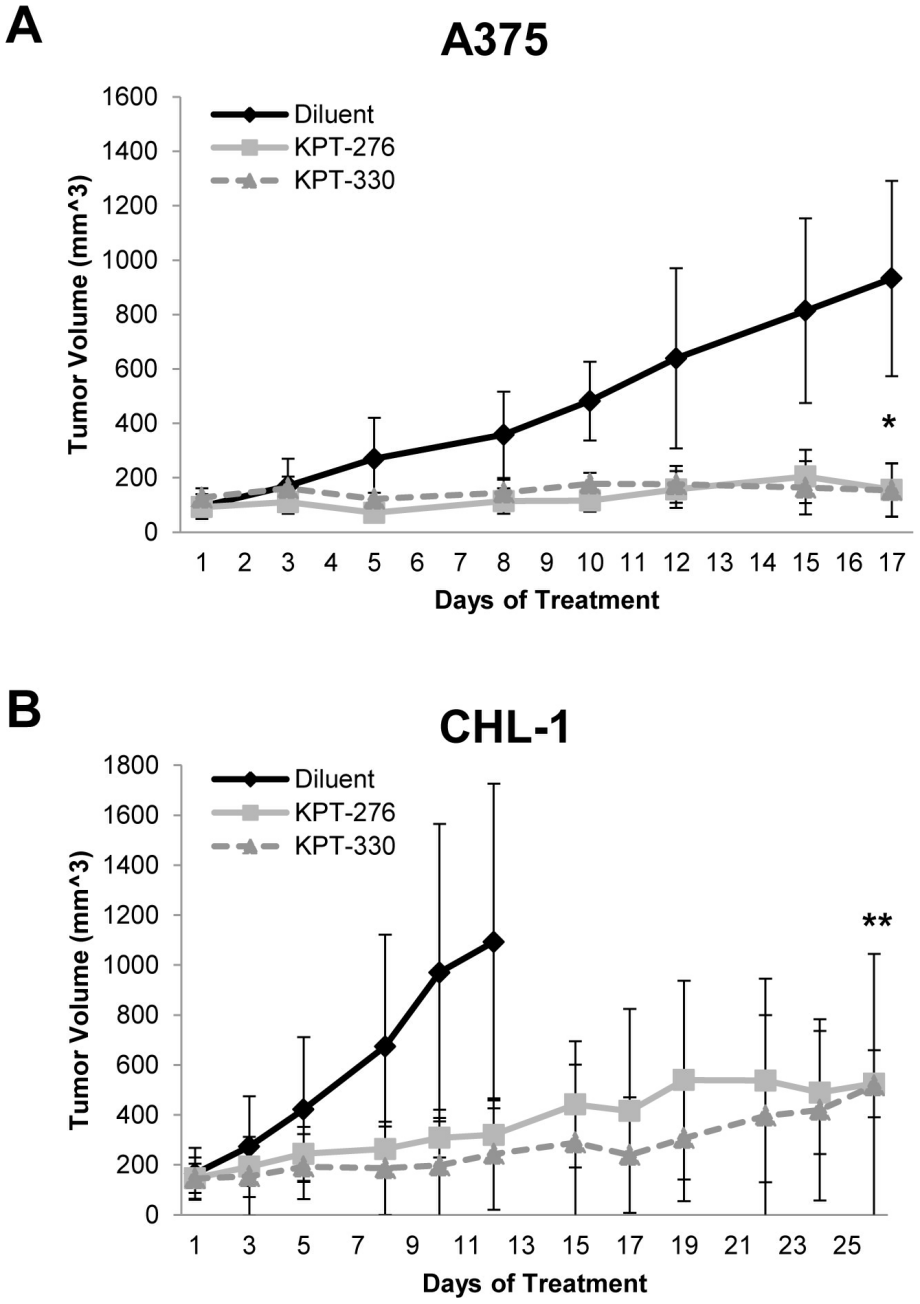
KPT-276 and KPT-330 reduce melanoma growth *in vivo*. Tumor xenografts of human *A*. A375 and *B*. CHL-1 melanomas in NOD/SCID mice were employed. Oral administration of KPT-276 or KPT-330 led to significant inhibition of tumor growth as compared to control mice treated with diluent alone (n = 6 for all groups). Statistical significance indicated by * =  p-values of <0.0001 for both KPT-276 and KPT-330 in A375, and ** =  p-values of 0.0087 and 0.0002 for KPT-276 and KPT-330 in CHL-1.

To evaluate the *in vivo* activity of SINE against human melanoma cells, tumor xenograft models of human A375 and CHL-1 melanoma cell lines were utilized. For these studies, tumor-bearing NOD/SCID mice were treated three times weekly with the orally bioavailable KPT-276 or KPT-330 SINE compounds or vehicle control. Those two, like the other SINE compounds, share the same chemical “warhead” (XPO1 selectivity) as KPT-185, but differ in their side chains, leading to improved *in vivo* pharmacokinetics (Fig. 4A). As expected, both KPT-276 and KPT-330 displayed *in vitro* effects against melanoma cells that were comparable to KPT-185 (Fig. 4B−C). When administered orally to CD1 mice at 10 mg/kg, KPT-276 and KPT-330 displayed very favorable pharmacokinetic properties. The bioavailability of KPT-276 and KPT-330 was roughly >90% and 60%, respectively. At Tmax (1 hr for KPT-276 and 0.5 hr for KPT-330) both compounds reach levels equal to roughly 2.5 micromolar (personal communication, Karyopharm Therapeutics, Inc.). The KPT-276 compound also significantly inhibited growth of both the human A375 (p<0.0001) and CHL-1 (p = 0.0087) melanoma cell lines as compared to control mice treated with diluent alone (Fig. 5A and 5B). Treatment with the KPT-330 compound resulted in similar efficacy, where the *in vivo* growth of human A375 and CHL-1 melanoma xenografts were significantly inhibited (p<0.0001 and p = 0.0002 respectively). To capture early changes in target modulation within melanoma cells, representative mice bearing A375 or CHL-1 established melanomas were treated for one week (four treatments total), and tumors were harvested for histologic analysis. Similar to *in vitro* experiments, these studies revealed a trend towards increased levels of nuclear TP53 and pMAPK in the KPT-276 treated A375 xenografts (Fig. S3A). In CHL-1 xenografts, there was a high basal expression of TP53, which did not change upon exposure to KPT-276. These tumors also displayed very limited pMAPK staining in both diluents and treatment groups (Fig. S3B). No significant changes in Ki67 staining were observed in tumors from vehicle or KPT-276 treatment groups for either cell line. Although limited conclusions can be drawn from a limited sample size, overall, these results were consistent with those observed *in vitro* using these same cell lines for each protein of interest, as CHL-1 cells typically displayed less abundant pMAPK as compared to A375 (Fig. 3B). All mice tolerated drug as indicated by minimal change in their body weight during treatment (Fig. S2).

## Discussion

In the present report, we demonstrate that elevated levels of XPO1 protein are present in primary as well as metastatic melanoma as compared to nevi. In addition, novel small molecule inhibitors of XPO1-mediated nuclear export displayed potent anti-proliferative and pro-apoptotic effects on human melanoma cell lines. These inhibitors promoted nuclear accumulation of TP53, pMAPK, and upregulation of CDKN1A within the cell. Potent *in vivo* cytostatic activity in two human xenograft models of melanoma was also observed at well tolerated doses. These data suggest that inhibition of nuclear export using oral SINE compounds represents a potential therapeutic target that deserves further clinical investigation.

XPO1 controls the nuclear export of ∼220 proteins, the majority of which have canonical “leucine rich” hydrophobic nuclear export sequences [34], [35]. This nuclear exporter also controls the export of mRNAs such as COX2 [36] and iNOS [37]. Interestingly, while there are six other known nuclear export proteins (XPO2-7), XPO1 is the *sole* nuclear exporter for the major tumor suppressor and growth promoting proteins [14]. XPO1 inhibition leads to cell cycle arrest [38] whereby cells with significantly damaged genomes, e.g., neoplastic cells, undergo apoptosis, while normal cells will re-enter the cell cycle when the XPO1 blockade has been relieved [39], [40]. In culture, normal cells tolerate prolonged XPO1 blockade (up to 4−5 days), whereas cancer cells will initiate an apoptotic program after 8−12 hours of exposure to SINE compounds. These observations suggest that XPO1 inhibition is more efficient at inducing apoptosis in neoplastic cells, consistent with the lack of chemotherapy-associated toxicities (e.g., myelosuppression, alopecia, mucositis) observed with SINE treatment. Data from our study are consistent with these observations and indicate XPO1 inhibition could effectively inhibit growth, by arresting cells in G1, and inducing apoptosis within a panel of human metastatic melanoma cell lines. While nearly 200 non-TSP are also substrates for XPO1, transient XPO1 inhibition with SINE compounds in prior studies did not appear to compromise normal cell function in prior studies [40]. In a similar manner, three separate primary human melanocyte cultures were less sensitive to XPO inhibition in the present study.

Despite the initial success of some molecular targeted therapeutics in melanoma, there remains a great need for other therapeutic agents that act in a manner independent of *BRAF* mutational status. XPO1 inhibition represents one potential approach to targeting melanoma cells of heterogeneous genotypes. Indeed, over 50% of melanoma patients have tumors with WT *BRAF* [41], and therefore do not respond to therapy against this target. Further, the development of drug resistance in *BRAF V600E* mutant melanomas can occur [42], [43], [44], often through MAPK reactivation [41], leaving a patient population with tumors that are in need of other effective therapeutic options. Recent publications on combined therapy with BRAF and MEK inhibitors (dabrafenib and trametinib respectively) highlighted significant increases in progression-free survival [45], suggesting the importance of these pathways in melanoma biology. Finally another recent report suggests XPO1 inhibitors may synergize with BRAF inhibitors to elicit antitumor effects against melanoma [26]. Together these data suggest that XPO1 inhibition is worthy of further investigation in the setting of advanced melanoma.

Nuclear accumulation of TP53 and downstream CDKN1A was observed following treatment with KPT-185 in the A375 melanoma cell line. These data suggest that activation of the TP53 pathway in response to cellular stress may be one operative mechanism of apoptosis following inhibition of nuclear export. Previous *in vitro* studies using the natural product XPO1 inhibitor leptomycin B (LMB) had demonstrated apoptosis in a TP53-dependent manner [20], [21], [46]. Another report from van der Watt *et al*. demonstrated that apoptosis was accompanied with increased TP53 and CDKN1A levels after XPO1 siRNA knockdown in cervical cancers [7]. In contrast, a representative cell line lacking wild type *TP53* was also responsive to XPO1 inhibition. This observation indicated that other pro-apoptotic mechanisms may be operative in response to this small molecule. For example, nuclear accumulation of pMAPK was observed following KPT-185 treatment. These data indicate nuclear export inhibition is a promising therapeutic approach that acts on multiple signaling pathways. Individual tumor lines may respond to inhibition of nuclear export via distinct mechanisms, including growth inhibition or apoptosis. These properties are likely influenced by numerous factors such as inherent differences in driver mutations across various types of tumors, constitutively activated signal transduction pathways, or soluble substances secreted from individual tumor types.

The oral administration of KPT-276 or KPT-330, structural analogs of KPT-185 with more favorable PK profiles, showed dramatic cytostatic activity *in vivo* against human melanoma xenografts. Mice were tolerant to the drug treatments, with minimal weight changes (Fig. S2) and no observed behavioral abnormalities, consistent with other *in vivo* reports (46−48). Immunohistochemical analysis of A375 tumors from a subset of mice treated with KPT-276 for a shorter duration also suggested that increased nuclear localization of both TP53 and pMAPK may also occur *in vivo*, whereas only moderate induction of these proteins occurs in CHL-1 tumors. This may be due to the fact that A375 has higher basal level of pMAPK, which has been observed to phosphorylate TP53 at the Ser15 residue and lead to increased stabilization and transactivation of TP53 [13], [47], [48], [49]. These data are consistent with the anti-tumor effects of SINE in other solid tumor as well as hematologic malignancy models that have been published previously [38], [39], [40], [50]. For example, treatment of chronic lymphocytic leukemia cells with SINE caused nuclear retention of FoxO3a, IκB, and TP53, resulting in marked anti-tumor activity [40]. In studies involving mantle cell lymphoma, IκBα was shown to accumulate in the nucleus, leading to down regulated NF-κB pathway activation [50]. KPT-185 treatment of acute myeloid leukemia (AML) cells led to TP53 and NPM1 entrapment and down-regulation of the growth-promoting kinases FLT3 and KIT [38]. Finally, KPT-330 treatment in human T-cell acute lymphoblastic leukaemia (T-ALL) cell demonstrated potent anti-tumor activities while sparing normal haematopoietic cells [39].

Our mechanistic findings show that SINE XPO1 antagonists induce antitumor activity against melanoma via effects upon cell growth and apoptosis. These effects were observed in melanoma cell lines of diverse genotypes, along with anti-tumor activity against both *BRAF* mutant and *BRAF* wild type cell lines. These data suggest that XPO1 inhibition is a reasonable and promising therapeutic approach against melanoma. Along with previous findings, our data support the ongoing evaluation of SINE in appropriate early-phase clinical trials for melanoma or other malignancies (clinicaltrials.gov number NCT01607905).

## Supporting Information

Figure S1 **Localization of TP53, CDKN1A, and pMAPK in melanoma cell lines with etoposide.** A375 and CHL-1 cell lines were treated with 20 µM of etoposide and harvested after 24 hours of treatment. Lysates were evaluated via immunoblot analysis. Lamin B and GAPDH serve as loading controls for nuclear and cytoplasmic compartments respectively. The data shown are representative of 2 to 3 independent biological experiments.
(TIF)

Figure S2 **Body weights of mice remained constant during oral treatment with KPT-276 or KPT-330.** Along with tumor measurements, the weight of the mice was determined throughout treatment (n = 6 for all groups).
(TIF)

Figure S3 **Immunohistochemical analysis of TP53, CDKN1A, pMAPK, and Ki67 in representative xenografts from mice bearing** ***A*** **. A375 tumors and** ***B*** **. CHL-1 tumors.** Representative images (scanned at 40× magnification) are shown for each tumor type following treatment with either diluent or KPT-276. *C*. Quantification of percent nuclear positivity for the proteins from *A*. and *B*. in the xenografts of KPT-276 treated mice (n = 3) compared to diluent treated mice (n = 2).
(TIF)
