# Supplementary figures and images for: Novel Small Molecule XPO1/CRM1 Inhibitors Induce Nuclear Accumulation of TP53, Phosphorylated MAPK and Apoptosis in Human Melanoma Cells
Source: PLoS One. 2014 Jul 24;9(7):e102983. doi: 10.1371/journal.pone.0102983 (PMC4109950; doi:10.1371/journal.pone.0102983)

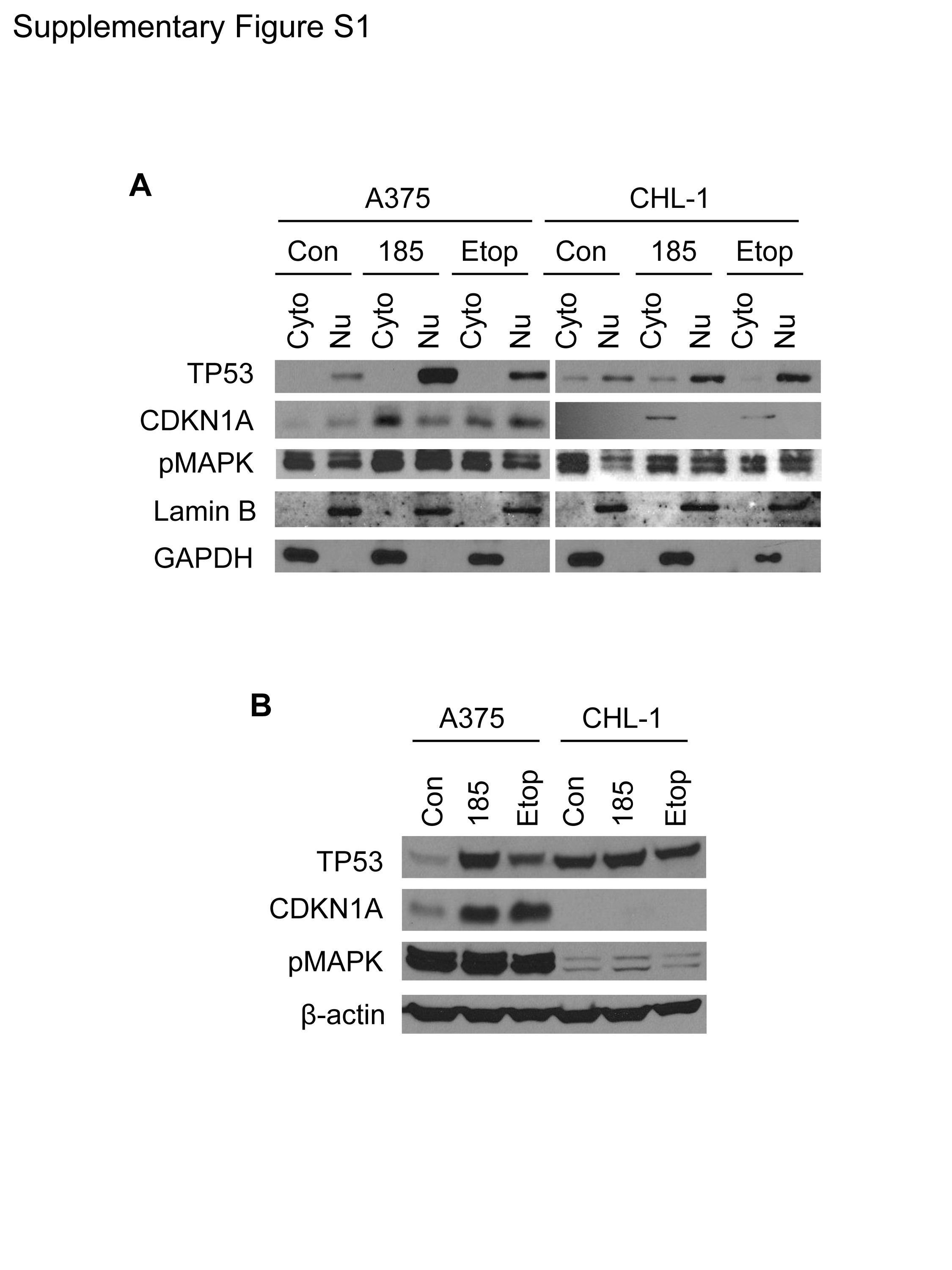

Supplement: Figure S1 — Localization of TP53, CDKN1A, and pMAPK in melanoma cell lines with etoposide. A375 and CHL-1 cell lines were treated with 20 µM of etoposide and harvested after 24 hours of treatment. Lysates were evaluated via immunoblot analysis. Lamin B and GAPDH serve as loading controls for nuclear and cytoplasmic compartments respectively. The data shown are representative of 2 to 3 independent biological experiments. (TIF) [file pone.0102983.s001.tif]

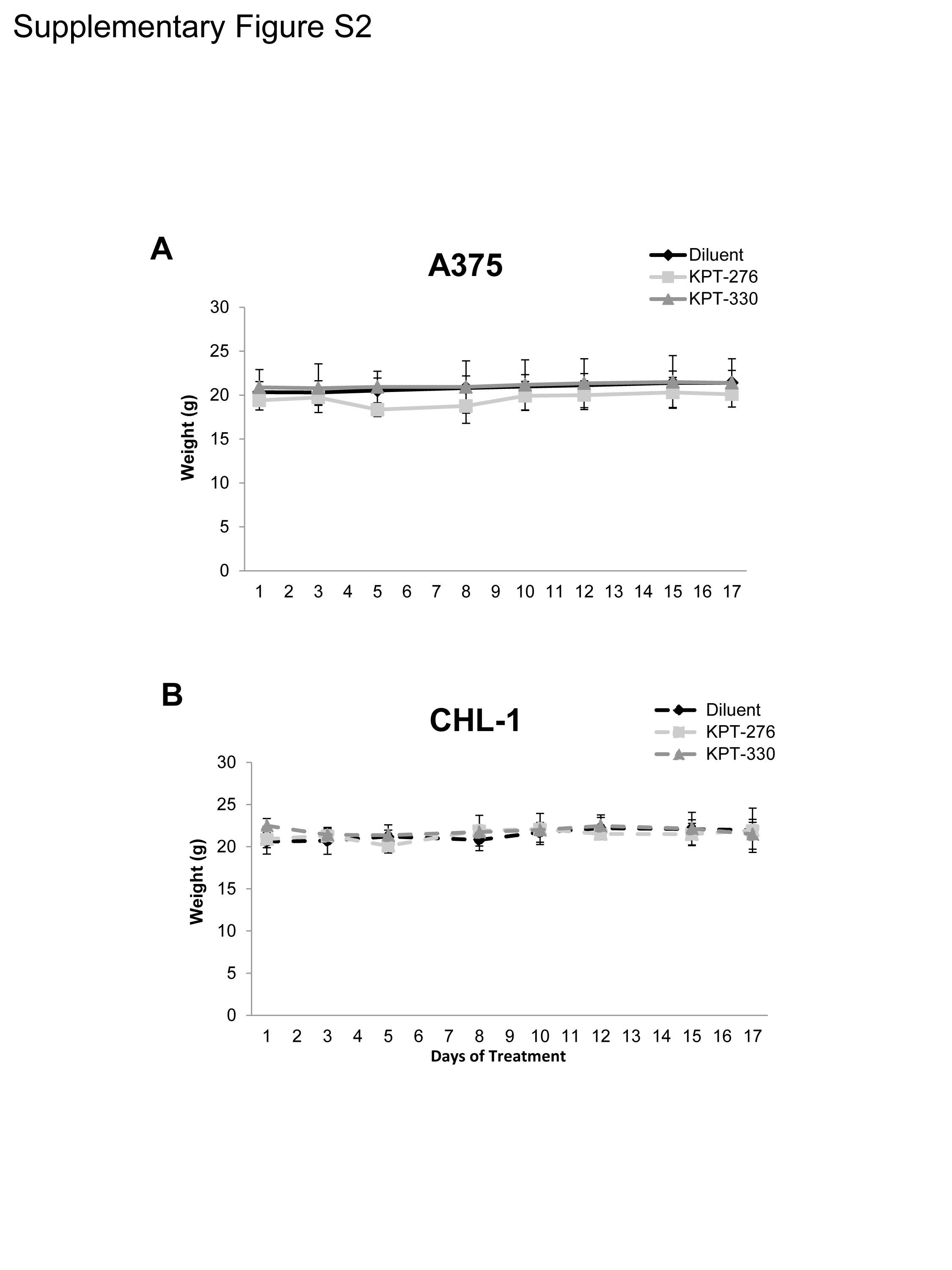

Supplement: Figure S2 — Body weights of mice remained constant during oral treatment with KPT-276 or KPT-330. Along with tumor measurements, the weight of the mice was determined throughout treatment (n = 6 for all groups). (TIF) [file pone.0102983.s002.tif]

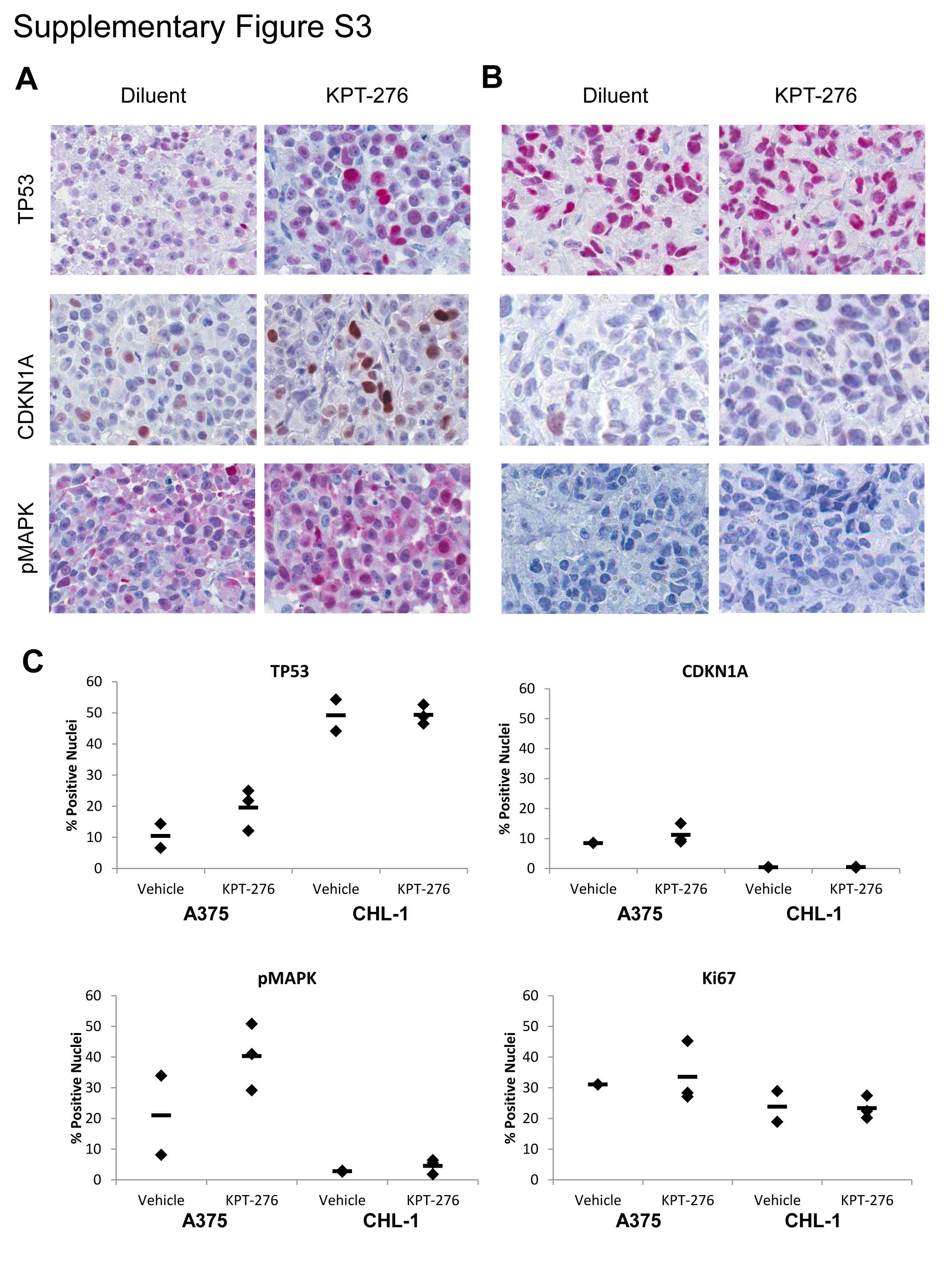

Supplement: Figure S3 — Immunohistochemical analysis of TP53, CDKN1A, pMAPK, and Ki67 in representative xenografts from mice bearing A . A375 tumors and B . CHL-1 tumors. Representative images (scanned at 40× magnification) are shown for each tumor type following treatment with either diluent or KPT-276. C. Quantification of percent nuclear positivity for the proteins from A. and B. in the xenografts of KPT-276 treated mice (n = 3) compared to diluent treated mice (n = 2). (TIF) [file pone.0102983.s003.tif]
